# On the Time Course of Vocal Emotion Recognition

**DOI:** 10.1371/journal.pone.0027256

**Published:** 2011-11-07

**Authors:** Marc D. Pell, Sonja A. Kotz

**Affiliations:** 1 School of Communication Sciences and Disorders, McGill University, Montréal, Québec, Canada; 2 Research Group “Neurocognition of Rhythm in Communication”, Max Planck Institute for Human Cognitive and Brain Sciences, Leipzig, Germany; University of Minnesota, United States of America

## Abstract

How quickly do listeners recognize emotions from a speaker's voice, and does the time course for recognition vary by emotion type? To address these questions, we adapted the auditory gating paradigm to estimate *how much* vocal information is needed for listeners to categorize five basic emotions (anger, disgust, fear, sadness, happiness) and neutral utterances produced by male and female speakers of English. Semantically-anomalous pseudo-utterances (e.g., *The rivix jolled the silling*) conveying each emotion were divided into seven gate intervals according to the number of syllables that listeners heard from sentence onset. Participants (*n* = 48) judged the emotional meaning of stimuli presented at each gate duration interval, in a successive, blocked presentation format. Analyses looked at how recognition of each emotion evolves as an utterance unfolds and estimated the “identification point” for each emotion. Results showed that anger, sadness, fear, and neutral expressions are recognized more accurately at short gate intervals than happiness, and particularly disgust; however, as speech unfolds, recognition of happiness improves significantly towards the end of the utterance (and fear is recognized more accurately than other emotions). When the gate associated with the emotion identification point of each stimulus was calculated, data indicated that fear (M = 517 ms), sadness (M = 576 ms), and neutral (M = 510 ms) expressions were identified from shorter acoustic events than the other emotions. These data reveal differences in the underlying time course for conscious recognition of basic emotions from vocal expressions, which should be accounted for in studies of emotional speech processing.

## Introduction

Empirical descriptions of the cognitive system for recognizing vocal emotion expressions in speech, or *emotional prosody*, are now accumulating rapidly. Emotional prosody refers to how speakers communicate emotion (intentionally or unintentionally) by modifying acoustic attributes of their voice, and how these cues are perceived and recognized by listeners. The neurocognitive system for analyzing emotions from prosody is thought to be functionally distinct from related mechanisms which process linguistic speech information [1], [2] and other socially-relevant meanings of the voice, such as the sex or identity of the speaker [3], [4]. Like facial expressions of emotion (e.g., [5], there is evidence that vocal emotion expressions are perceived and recognized in a categorical manner during speech processing [6], [7], [8]. These findings fit with the view that, due to the biological and social significance of co-ordinating emotional behaviour in human communication, there is a limited set of basic emotions which have discrete forms of expression in the face as well as the voice [9], [10], [11]. This hypothesis is supported by evidence that vocal expressions of anger, disgust, fear, sadness, and happiness/joy can be accurately recognized when listening to a foreign language [12], [13], [14], [15], implying that these emotions possess discrete acoustic-perceptual properties in the voice which manifest in similar ways across languages [16].

What is still poorly understood in this literature is the temporal evolution, or relative *time course*, for recognizing discrete emotional meanings from prosody. That is, how and *when* do changes in the speech stream lead to familiarity and actual recognition of a speaker's emotional state? And is the time course for emotion recognition in the vocal channel similar for all basic emotions? These questions touch upon the very nature of how emotions are encoded in the vocal channel, and how representational details of these events are presumably activated to promote recognition of discrete emotions as speech unfolds.

Decoding emotions in speech includes independent stages for extracting sensory/acoustic features, for detecting meaningful relations among these features over time, and for conceptual processing of the acoustic patterns in relation to emotion-related knowledge held in long-term memory [17]. At the stages of conceptual processing, it has been argued that emotion-specific knowledge associated with basic emotions is stored as separate units in an associative memory network (e.g., [18], [19], [20]). These representations can be activated by prototypical acoustic or sensorimotor features associated with the emotion expression when encountered in the auditory or visual modalities [7]. Data show that emotion-specific meanings in speech are registered implicitly and automatically by vocal cues [21], [22], [23], [24], presumably after a series of more basic appraisals of the incoming event to determine its valence, potency, and other affective details which contribute to emotional knowledge [25].

While informative, these details do not reveal *how much* information is needed to recognize discrete emotions from vocal attributes of speech. Understanding the issue of timing is critical to specify the cognitive system devoted to vocal emotion processing, since vocal expressions are ubiquitously dynamic, dictated by their temporal structure (and unlike facial expressions, cannot be tested in a static form). Vocal emotion expressions are differentiated by acoustic *patterns*, as opposed to individual acoustic parameters [26], [27], [28]; listeners attend to both absolute and relative settings (mean + variation/range) of a speaker's vocal pitch, loudness, and how other acoustic features change over time to form discrete impressions about the speaker's emotion as speech unfolds (see [29] for a detailed overview). The dynamic interplay of pitch and speaking rate for recognizing emotions has been emphasized by several studies [30], [31], [32]. For example, based on a recent comparison of English, German, Hindi and Arabic, there is evidence that fear tends to be communicated with a relatively fast speaking rate, high pitch, and moderate pitch variation, whereas sadness is expressed with a slow speaking rate, low pitch, and little pitch variation; acoustic differences in pitch and timing differentiate vocal expressions of anger, disgust, happiness, surprise and neutral utterances as well [16].

Thus, to characterize when vocal emotions are registered and become ‘accessible’ for recognition processes, one must consider the *time* that listeners are exposed to fluctuations in pitch, loudness, and other representative acoustic cues which specify their meanings in speech. (It is assumed that acoustic patterns progressively activate conceptual details which lead to familiarity and recognition of the speaker's emotion state; for a recent discussion, see [33]). Given variability in the underlying temporal properties of vocal expressions, it is possible that discrete emotions in the voice unfold at different rates, and are thus recognized at different points in time, as implied by recent priming data [34]. For example, recognition times might vary according to the biological significance of the signal to initiate a behavioural response, such as vocal signals which indicate threat [35], [36], [37]. Unfortunately, while studies have investigated how quickly basic affective details of vocal expressions, such as their intensity or valence, are registered (within the first 100–200 ms [17], [38]), there is only a small literature which informs the potential time course for recognizing discrete emotional meanings conveyed by speech prosody.

Using evidence from event-related brain potentials (ERPs), Paulmann and colleagues [38], [39] have tested when emotional meanings of prosody are *implicitly* detected, and whether these meanings can be differentiated during on-line speech processing. Paulmann and Kotz [38] presented vocal expressions in German to 31 listeners who performed a probe (word) verification task while implicitly processing emotional prosody. A systematic reduction in P200 amplitudes was observed for six different emotional expressions (anger, disgust, fear, sadness, happiness, pleasant surprise) when each emotion was compared to corresponding neutral sentences, although there was no modulation of the P200 component when the six emotions were directly compared. These findings suggest that processes involved in the acoustic extraction of vocal parameters, which highlight the emotional *salience* of vocal expressions (i.e., as emotional versus non-emotional), occur within the first 200 milliseconds following speech onset; any differentiation of discrete emotional meanings must therefore occur at a somewhat later stage of analysis [38].

This conclusion fits with recent data reported by Paulmann and Pell [39]; in that study, 24 English participants heard excerpts of emotional pseudo-utterances, lasting either 200 ms or 400 ms in duration, followed by a facial expression that was emotionally congruent or incongruent with the vocal prime stimulus. Participants made a facial affect decision about the face target [7]. Results indicated that listening to vocal expressions of anger, fear, sadness, or happiness produced a classically distributed N400 effect on the face when congruent versus incongruent trials were compared across emotions, in the 400 ms condition but not in the 200 ms condition. Since N400 differences in this context index whether underlying meanings of the prosody and face are the same emotion, these findings suggest that listening to only 200 milliseconds of emotional speech does not sufficiently activate emotional meanings from prosody, although these meanings are implicitly recognized when vocal expressions lasted 400 ms [39]. Related studies have also linked amplitude differences in the N300 component to initial conceptual processing of vocal emotional stimuli [40], [41]. These results begin to narrow the time window in which emotional meanings of the voice are implicitly detected to around 300–400 milliseconds of speech [4], [17], [40]. Interestingly, this general time window fits with recent *behavioural* measures reported by Pell [42]; when happy, sad, or neutral pseudo-utterances spoken in English were gated from the onset of the sentence to last 300, 600, or 1000 milliseconds in duration, emotional priming of a congruent face was only observed when vocal cues endured for 600 or 1000 ms, but not when the prime was presented for only 300 ms in duration. These results imply that emotion-specific details about vocal expressions are registered and attain the necessary threshold to prime an emotionally congruent face in the time window of 300–600 milliseconds following speech onset [34], [42]. This roughly approximates the time window where discrete emotions appear to be recognized based on the ERP evidence cited above.

However, the timing of *implicit* effects of emotional prosody, as inferred from priming or other on-line measures, may not directly correspond to when this knowledge is accessible for conscious processing and explicit decisions about a speaker's emotion. There is a long-standing tradition for researchers to assess emotion recognition using explicit emotion judgements, typically forced-choice tasks, where participants must *name* the emotion conveyed by the stimulus from a set of alternatives [16], [43], [44], [45]. Forced-choice tasks index processing stages leading to the activation of emotion-related knowledge from vocal cues, as well as procedures for strategically accessing and comparing activations of the input for their presumed ‘goodness-of-fit’ with emotional language categories (see [46] for a methodological discussion). While forced-choice methods have known limitations (see [47]), this approach informs much of what we know about how vocal emotions are recognized in speech and still provides constructive insights. For example, it is clear that specific emotions, such as sadness and anger, are recognized very well from prosody, whereas emotions such as disgust (or surprise) are recognized relatively poorly from vocal cues [16], [26], [48]. While these investigations do not speak to the time course of emotion recognition, they firmly establish that when listeners are exposed to relatively long speech samples (i.e., sentences of approximately 1–2 seconds in duration), not all emotions are recognized equally well.

New endeavours are needed to document the time course of vocal emotion recognition, especially data which can be compared to existing knowledge derived from forced-choice tasks (where “recognition” can be defined as the ability to consciously reflect on and *categorize* vocally-expressed emotions). One approach that has been used successfully to estimate the temporal course of operations leading to recognition of auditory events is the gating paradigm [49]. Traditionally, this technique has been used to investigate processes of lexical retrieval/word recognition and phoneme identification (e.g., [50], [51], [52]). Recently, it has been adapted to test how listeners narrow-in on discrete emotional meanings conveyed by music [53] or in speech [54], [55]. In gating studies, auditory “gates” are constructed as a function of specific time increments, or linguistic units of spoken language, and then presented to listeners in segments of increasing duration starting at the beginning of the relevant stimulus, where the last gate usually corresponds to the entire stimulus event (see [56] for an overview of design issues). This task, which many consider to be a sensitive, on-line measure of spoken language processing [57], yields both *qualitative* information about how accurate and confident listeners are about the presence of discrete emotions at each gate interval, and *quantitative* information about how much acoustic variation is needed to achieve different levels of accuracy, and ultimately, to “isolate” discrete emotions in the speech stream. For example, researchers can estimate the “identification point” of specific target meanings by locating the gate at which the target is accurately recognized by a participant without further changes at longer gate durations for the same stimulus [50], [51].

The use of gating to estimate the time course of *emotion* recognition in speech is still rare. In an old study, Pollack et al. [58] reported that 60 ms utterances gated from sentence onset yielded good recognition of eight expression “modes”,including fear and happiness, when listeners categorized these meanings from a fixed set of response alternatives; unfortunately results of this study are difficult to interpret due to sparse reporting of methodological details and data, and because many of the expression modes of interest (e.g., “objective question”, “confidential communication”) do not fall within an accepted theoretical framework about emotions). Similary, a gating study by Audibert and colleagues [54] reported differences in how well listeners identify eight affective expressions—anxiety, disappointment, disgust, disquiet, joy, resignation, sadness, satisfaction—from monosyllabic words gated at different locations in the vowel or consonant. While their data imply that emotions such as “joy” and “disgust” are often recognized less accurately than many other affective modes, the authors caution about the small number of stimuli presented in their study, and again, these patterns do not reflect the presumed effects of discrete emotion categories on vocal emotion recognition over time.

Recently, Cornew, Carver and Love [55] reported two experiments in which they gated pseudo-utterances—Jabberwocky sentences ranging in duration from 1.6–4.4 seconds—which had been produced by a single actress to express anger, happiness, or neutrality. Stimuli were gated successively in 250 millisecond increments and then categorized by a group of listeners in a three forced-choice task (Expt 1) or in a discrimination task (Expt 2). Following Grosjean [49], the “isolation point” for each emotion (i.e., gate where participants correctly identified the target emotion and did not change their response at longer intervals) was calculated to determine whether there was an advantage to recognize positive, negative, or neutral prosody. Results indicated that the isolation point differed significantly for each emotion as sentences unfolded, with a bias for recognizing neutral sentences quickly and accurately (mean isolation time across participants = 444 ms) followed by angry (*M* = 723 ms) and finally happy (*M* = 802 ms) sentences. The authors concluded that there may be an advantage for recognizing neutral, rather than emotional, content during emotional prosody processing [55]. However, given that very few emotions were investigated in this study and that speech stimuli were not fully controlled in certain respects (i.e., in terms of item length, the effects of lexico-semantic content across emotion conditions), a more rigorous investigation of this nature is needed.

The purpose of the present study was twofold: to document how the recognition of discrete emotions in the voice evolves at different points of juncture as spoken utterances unfold; and to estimate the time at which each emotion is recognized in the utterance (i.e., to compute its “emotion identification point”). A secondary goal was to characterize some of the major acoustic features which differentiate vocal expressions of basic emotion at their precise emotion identication point. To accomplish these objectives, we employed the auditory gating paradigm and focused our analyses on a core set of frequently studied basic emotions—anger, disgust, fear, sadness, happiness—as well as neutral utterances. Following previous researchers [12], [26], [36], [55], we presented emotionally-inflected pseudo-utterances to ensure that listener judgements of emotion were based only on prosodic cues, rather than linguistic information which could bias emotional meanings during speech processing. Contrary to Cornew et al. [55] who divided utterances into 250 millisecond time intervals, we defined auditory gates according to a major linguistic unit of spoken utterance, the syllable boundary [59], to capture how emotional meanings unfold over the course of an utterance. Defining gates according to linguistic units, rather than time, allowed us to rigorously control the linguistic-phonetic content of speech information presented at each gate across emotion conditions, given that differences in speaking rate are one of the primary cues for recognizing vocal emotions [29]. Since all sentences were seven syllables in length, items could be gated from sentence onset for presentation in seven distinct gate conditions, where listeners always identified the emotion being conveyed by the speaker in a six forced-choice response format.

Based on the literature, we hypothesized that recognition of each emotion would systematically increase at successive gate intervals, and that accuracy at the longest gate interval (i.e., full utterance) would vary by emotion type (e.g., accuracy should be relatively good for anger and sadness and poor for disgust). However, we expected that emotion-specific differences in recognition would be observed at much earlier gate durations in the utterance, and that the “identification point” for many emotions would begin to emerge for gated stimuli lasting more than 300 milliseconds and less than 600 milliseconds in duration [17], [39], [40], [42]. No strong predictions could be made about whether the identification point would be similar for all basic emotions, although we anticipated that not all emotion types would unfold at the same rate in speech [34] and that neutral utterances would be recognized more quickly than anger or happiness [55]. When acoustic measures are examined, we expected that differences in pitch (mean + variation) would play an especially important role in predicting how and *when* discrete emotions are accurately recognized in speech, based on their emotion identification point.

## Methods

### Ethics Statement

This study was ethically approved by the McGill Faculty of Medicine Institutional Review Board in accordance with principles expressed in the Declaration of Helsinki. Informed written consent was obtained for each participant prior to their involvement in the research.

### Participants

Forty-nine participants (25 male, 24 female) completed the study after responding to an electronic advertisement posted at McGill University. Participants averaged 22.3 years in age (SD = 4.0) and had completed an average of 15.9 years of formal education (SD = 2.3). All participants had learned English from birth and reported no hearing difficulties.

### Stimulus recording and selection

The stimuli were digital recordings of emotionally inflected “pseudo-utterances” produced by native speakers of Canadian English, selected from a perceptually-validated inventory. As described elsewhere in full [16], this inventory consists of 30 unique pseudo-utterances (e.g., *The rivix jolled the silling*) which were each produced to express seven different emotion types (anger, disgust, fear, sadness, happiness, pleasant surprise, neutral). The emotional expressions were posed (simulated) by two male and two female lay actors in a way that was natural to the speaker as part of an emotion elicitation procedure, followed by a perceptual validation study (see [60], [61] for similar approaches). Pseudo-utterances were used because they can be readily produced by speakers to convey emotions in the voice and strongly ressemble the listeners' native language, but they limit meaningful emotion-related cues to prosody [12], [26], [36]. As reported by Pell et al. [16], perceptual data were gathered on each emotional utterance by presenting them to 24 English-speaking listeners; for each item, listeners first identified the emotion of the speaker from the seven alternatives (forced-choice format), and in a secondary judgement, they rated the intensity of the emotion expressed along a five-point Likert scale. These data were used to select a subset of emotional exemplars which were highly representative of each emotion for use in the present study.

For this study, we selected emotions in our inventory for which there is high agreement about their status as basic emotions with discrete forms of expression in the face and voice: anger, disgust, fear, sadness, and happiness/joy [62]. Surprise was excluded for this reason, and because surprise utterances are difficult to simulate experimentally [16], meaning that our database contained relatively few ‘good’ exemplars that would allow us to control for other stimulus features of items selected for the gating experiment. Corresponding neutral utterances were also included to establish a context for interpreting responses pertaining to the five basic emotions, resulting in a total of six emotion ‘types’ in the experiment. For each emotion type, 24 distinct items (6 per speaker) with the highest recognition rates in the validation study were selected, while controlling for differences in the linguistic-phonetic structure/length of items across emotion types (which could affect timing measures and the value of gate durations independent of emotion). All stimuli were seven syllables in length and each pseudo-utterance appearing in the experiment had been successfully produced by one of the four speakers to convey all six emotional meanings at a perceptually reliable consensus level (minimum 60% correct emotion recognition for the listener group in the validation study). These controls ensured that the linguistic composition of items which “carried” vocal cues to emotion was identical across emotion conditions. Within each emotion condition, the serial position of stressed syllables in the selected utterances was also controlled, since English is a stress-timed language, and stressed vowels could present local opportunities for speakers to modify acoustic cues in the service of emotion [27]. For all stimuli, the initial stressed syllable was always the second syllable of the pseudo-utterance; the second stressed syllable in the utterance varied, but fell in equal numbers on the fourth, fifth, or six syllable of the pseudo-sentence (this was fully balanced across emotions and speakers). To control for gross perceptual differences in the loudness of stimuli produced by different speakers in the experiment, the peak amplitude of all utterances was normalized to 75 dB. In total, 144 items (6 emotions×24 items) were selected for manipulation into different gate durations. The mean consensus of the listener group for the selected items, based on presentation of the full utterance, was high for all emotions: anger = 86%, disgust = 73%, fear = 89%, sadness = 90%, joy = 81%, and neutral = 81%. These values represent target recognition of at least five times chance expectation (14.3%) in the validation study [16].

### Gate construction

To document how discrete emotions unfold over the course of an utterance, we defined our gate increments according to the duration of each syllable of 7-syllable pseudo-utterances. This produced seven distinct gate duration intervals in the experiment, where items presented in Gate7 were always the unaltered pseudo-utterances from our inventory which were chosen for being good exemplars of each emotion category. Each of the 144 tokens was edited using Praat speech analysis software to produce six new stimuli which varied in the number of syllables presented from sentence onset (Gate1 to Gate7, where the numeral indicates the number of syllables presented to the listener from sentence onset). While sentence onset was uniform for each item across gate conditions (defined by the actual speech onset), each gate condition had a distinct offset as defined by the corresponding syllable boundary (this location was marked by auditory and visual inspection of the waveform). This process culminated in 144 distinct items which could be presented in each of 7 gate duration conditions (1008 items total).

To provide background data on our stimuli, each of the 1008 items was analyzed acoustically in Praat to summarize major acoustic parameters of the emotional exemplars presented in each gate duration condition; these data are furnished in Table 1. Since an equal number of identical items produced by each of the four speakers contributed to each emotion condition, raw measures of fundamental frequency (f0) provide an accurate description of emotion-related differences for this stimulus set. Table 1 shows that there were marked differences in the overall duration of exemplars conveying each emotion (ranging from disgust (*M* = 2126 ms) to fear (*M* = 1270 ms)), which reflect known tendencies in how these emotions are communicated in speech [29], [63]. Once these items were gated, this meant that the mean syllable duration also varied as a function of emotion: anger = 257 ms, disgust = 304 ms, fear = 182 ms, sadness = 263 ms, happiness = 238 ms, and neutral = 219 ms. Additional acoustic measures are reported in Table 1.

**Table 1:** Acoustic features of the experimental stimuli (measured from sentence onset for each cell).

		Gate Duration (# syllables)						
Measure	Emotion	G1	G2	G3	G4	G5	G6	G7
Duration	Anger	182	563	789	1033	1226	1467	1799
(ms)	Disgust	183	625	867	1155	1412	1705	2126
	Fear	119	363	491	649	790	979	1270
	Sadness	198	580	781	1026	1227	1478	1839
	Happiness	159	518	690	898	1080	1317	1665
	Neutral	150	444	649	852	1017	1218	1534
Speech Rate	Anger	5.5	3.6	3.8	3.9	4.1	4.1	3.9
(syllables/s)	Disgust	5.5	3.2	3.5	3.5	3.5	3.5	3.3
	Fear	8.4	5.5	6.1	6.2	6.3	6.1	5.5
	Sadness	5.1	3.4	3.8	3.9	4.1	4.1	3.8
	Happiness	6.3	3.9	4.3	4.5	4.6	4.6	4.2
	Neutral	6.7	4.5	4.6	4.7	4.9	4.9	4.6
f0Mean	Anger	223	232	225	218	215	211	204
(Hz)	Disgust	179	179	182	178	176	170	170
	Fear	314	287	284	277	272	270	260
	Sadness	199	196	192	186	183	180	184
	Happiness	218	220	220	214	207	198	196
	Neutral	160	159	166	163	162	157	154
f0Range	Anger	40	119	151	168	181	182	212
(Hz)	Disgust	34	93	128	152	189	185	223
	Fear	28	89	97	132	130	132	182
	Sadness	22	74	84	107	110	124	196
	Happiness	26	86	95	133	131	159	196
	Neutral	13	32	58	82	103	128	122

### Experimental procedure

Each participant was tested individually in a quite laboratory during a single session lasting approximately 1.5 hours. Stimulus presentation was controlled by a laptop computer running Superlab 4.0 software (Cedrus, USA). To mitigate potential artefacts such as response perseveration [56], stimuli representing each gate duration were presented in a duration-blocked format which always began with Gate1 and ended with Gate7. At each gate duration, pseudo-utterances representing the six emotions were fully intermixed and presented in a unique random order across participants. During the experiment, each utterance was played a single time over headphones at a comfortable listening level; after listening to the item, the participant was instructed to make two judgements in sequence. First, the participant was required to name which of the six target emotions was being expressed by the speaker by selecting the corresponding emotion term from a printed list on the computer screen; the precise emotion labels used were *anger*, *disgust*, *fear*, *sadness*, *happiness*, and *neutral* (the positioning of emotion labels on the screen was randomized and varied within the participant group). Once the emotion of the voice was categorized, a seven-point rating scale appeared on the computer screen and the participant rated how confident they were about their emotional judgement for that instance (where 1 = “very unsure” and 7 = “very sure”). All data were recorded automatically by the computer and trials were separated by a two second interval. Each block was preceded by a series of 10 practice trials (which did not appear in the experiment) which accustomed the participant to the sound of the pseudo-utterances, the length of the stimuli, and the response format. Participants were informed in advance that the sentences were not supposed to “make sense” and that they should attend to the emotion conveyed by the speaker. Participants were instructed to choose the emotion that “best fit” what they heard whenever in doubt about the speaker's emotion. Participants received $25 CAD after completing the experiment.

### Statistical analyses

Analyses were performed on data from 48 participants (24 male, 24 female); one of the original male participants was excluded due to a failure to comply with task goals. First, analyses examined how accurately vocal expressions of each emotion were recognized by the 48 participants at each gate duration, for the raw hit rates (in percent correct) and once these data were corrected for individual biases in the use of particular emotion response categories (i.e., through the computation of “Hu scores” [64]). These comparisons reveal how accurately discrete emotions are recognized from prosody for identical linguistic units processed over the course of an utterance. At a second stage, analyses sought to specify the “emotion identification point” for each gated stimulus, by calculating which of the seven gate intervals yielded correct identification of the target emotion without subsequent changes at longer gate intervals of the same stimulus [50]. This analysis provides information on the number of syllables and corresponding *time* needed to accurately recognize discrete emotion meanings, and how this differs by emotion type. Finally, the estimated emotion identification point of each exemplar was transformed into its corresponding acoustic measures to infer which parameters may be necessary for recognizing emotions, and how these differ by emotion type. All comparisons were tested using repeated measures ANOVAs (*p<*.01). The size of significant effects was characterized by partial Eta-squared (*ŋ^2^_Partial_*) and they were elaborated, when relevant, using Tukey's (HSD) post hoc comparisons (*p*<.01).

## Results

### Recognition of discrete emotions by gate duration

Table 2 supplies the mean correct target responses (% correct) and mean confidence ratings (scale of 1–7) of the 48 participants when judging utterances representing each emotion type, at each gate duration interval.

**Table 2:** Mean accuracy (% target recognition) and confidence ratings (scale of 1–7) for 48 listeners who judged utterances representing each emotion type, according to the gate duration.

		Gate Duration (# syllables)						
Measure	Emotion	G1	G2	G3	G4	G5	G6	G7
Accuracy	Anger	39.3(20.6)	60.2(27.4)	66.7(24.8)	73.5(25.5)	75.0(25.7)	78.1(26.1)	78.9(24.7)
	Disgust	13.9(9.8)	22.7(11.6)	31.3(14.5)	39.4(15.3)	46.4(14.4)	55.3(13.9)	69.1(13.6)
	Fear	31.7(12.8)	53.2(21.1)	68.7(22.1)	74.7(18.3)	79.5(17.9)	83.2(14.9)	86.5(13.7)
	Sadness	62.3(22.1)	76.6(15.8)	79.5(16.0)	81.9(15.5)	83.9(8.7)	86.0(9.7)	85.9(12.5)
	Happiness	12.6(10.8)	29.3(21.3)	40.8(23.9)	54.8(24.3)	68.5(20.5)	79.0(16.0)	87.1(13.4)
	Neutral	57.3(8.9)	74.1(8.9)	78.4(8.0)	82.0(9.3)	82.2(10.1)	85.6(8.5)	87.0(10.1)
ConfidenceRatings*	Anger	3.5(1.2)	4.8 (0.9)	5.1 (0.8)	5.4 (0.7)	5.7 (0.8)	5.9 (0.7)	6.2 (0.7)
	Disgust	3.5 (1.2)	4.3 (0.9)	4.5 (0.9)	4.7 (0.8)	5.0 (0.9)	5.2 (0.9)	5.7 (0.9)
	Fear	3.6 (1.2)	4.6 (0.9)	5.0 (0.9)	5.2 (0.8)	5.5 (0.7)	5.8 (0.7)	6.1 (0.6)
	Sadness	3.7 (1.2)	4.8 (0.9)	5.0(0.9)	5.1 (0.9)	5.5 (0.8)	5.7 (0.8)	6.1 (0.7)
	Happiness	3.3 (1.1)	4.3 (1.0)	4.5 (0.9)	4.9 (0.9)	5.2 (0.9)	5.6 (0.9)	6.1 (0.7)
	Neutral	3.3 (1.2)	4.3 (1.0)	4.6 (0.9)	4.9 (0.8)	5.2 (0.8)	5.6 (0.9)	6.0 (0.8)

Standard deviations are shown in parentheses.

*For correct target responses only.

#### (i) Accuracy measures

Inspection of the raw hit rates in Table 2 demonstrates that recognition of each emotion always improved at successive gate intervals, although there were marked differences in how accurately the six emotion expression types could be identified from (otherwise identical) pseudo-utterances at most time intervals. Even when participants heard only the first, unstressed syllable of an utterance (Gate 1), emotion-related accuracy differences were clearly evident, although these patterns tended to converge towards the end of the utterance (with the exception of disgust). Based on the raw hit rates, recognition of sadness and neutral expressions was notably more accurate than for the other emotions at early points of the utterance (between Gates 1–3); in contrast, happiness and disgust tended to be recognized less accurately than the other emotions at most time intervals in the utterance.

Statistical analysis of the accuracy data was performed on the *unbiased* hit rates [64] after correcting for individual bias in the frequency of response categories used at each gate interval for each of the 48 participants (response category usage across participants is provided in Table 3) . Here, H_u_ scores denote the unbiased proportion of correct responses observed for each of the six emotions at a given stimulus gate, where a score of zero reflects chance performance at that gate and a score of one reflects perfect performance. These data were transformed (arcsine) and then analyzed using a 6×7 ANOVA with repeated measures of Emotion (anger, disgust, fear, sadness, happiness, neutral) and Gate duration (1–7). The analysis yielded significant main effects for Emotion, *F* (5, 235) = 121.29., *p*<.0001, *ŋ^2^* = 0.72, and Gate duration, *F* (6, 282) = 386.51, *p*<.0001, *ŋ^2^* = 0.89, and the interaction of Emotion and Gate duration , *F* (30, 1410) = 17.67, *p*<.0001, *ŋ^2^* = 0.27.

**Table 3:** Mean proportion of emotional response category usage at each gate duration interval for the 48 listeners (includes both correct and incorrect target responses).

	Gate Duration (# syllables)						
Emotion	G1	G2	G3	G4	G5	G6	G7
Anger	0.16	0.16	0.15	0.16	0.15	0.15	0.14
Disgust	0.09	0.10	0.11	0.12	0.13	0.13	0.17
Fear	0.14	0.14	0.16	0.15	0.15	0.15	0.15
Sadness	0.26	0.27	0.25	0.24	0.22	0.21	0.18
Happiness	0.08	0.09	0.11	0.12	0.15	0.16	0.18
Neutral	0.27	0.24	0.22	0.21	0.20	0.20	0.18

Post hoc (Tukey's) elaboration of the interaction first looked at how recognition of each emotion evolved as a function of hearing incrementally more gates (syllables) of an utterance. Recognition of anger and neutral expressions improved significantly between all intervals from Gates 1 to 4, fear improved significantly between all intervals from Gates 1 to 6, and happiness improved significantly between every gate interval of the utterance (Gates 1 to 7). Recognition of sadness improved incrementally but these changes were only significant between Gates 1–2 and again between Gates 6–7. In contrast to the other emotion types, recognition of disgust improved only in the second half of the utterance, increasing significantly between all intervals from Gates 4 to 7.

**Figure 1:**
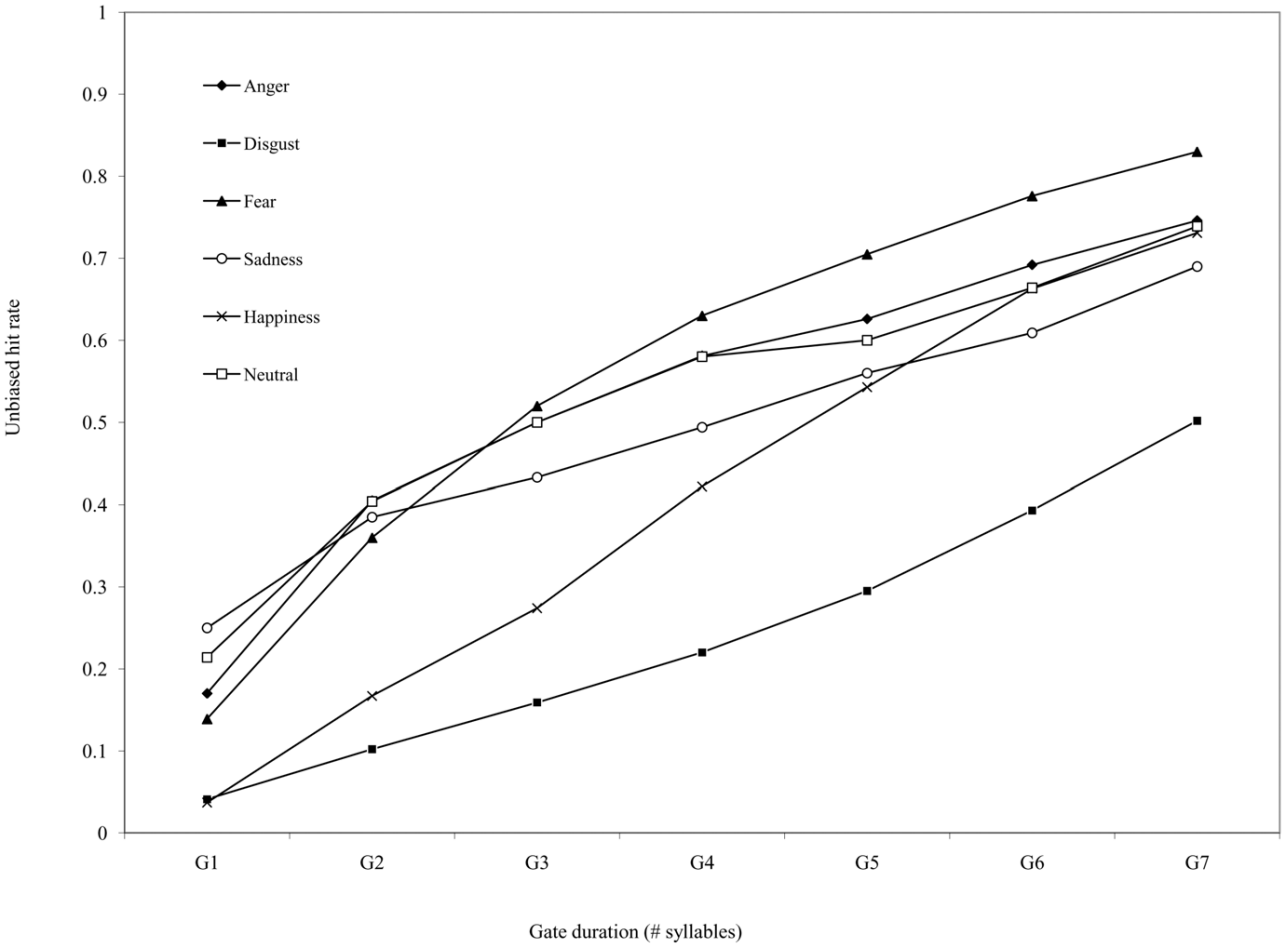
Mean unbiased accuracy of the 48 listeners to recognize utterance conveying each emotion, as a function of gate duration (number of syllables heard).

When recognition accuracy was compared directly across emotion types at each gate interval, several patterns of importance emerged. First, the data indicate that anger, sadness, fear, and neutral expressions were recognized with comparable accuracy between Gates 1 to 4; after this gate interval, vocal expressions of fear were always recognized significantly better than all other emotions (i.e., from Gate 5 to the end of the utterance). Second, the data show that happiness was recognized significantly less accurately than anger, fear, sadness, and neutral expressions up to Gate 5 in the utterance; however, with increased exposure to speech at later gate intervals (Gates 6 and 7), recognition of happiness did not significantly differ from anger, sadness, or neutral expressions (although all of these emotions were identified less accurately than fear). Finally, disgust was always recognized more poorly than all other emotions, except at Gates 1 and 2 where accuracy for disgust and happiness did not differ significantly. These patterns, which supply new information about how the recognition of discrete emotion expressions unfolds in spoken utterances, are illustrated in Figure 1.

To briefly explore whether participant sex influenced these findings, the 6×7 ANOVA was rerun with Sex (female, male) as a grouping factor in the analysis. There was no significant main effect of sex on accuracy in the experiment (p = .57), nor did sex influence performance as a function of Gate duration (p's>.26 for corresponding two- and three-way interactions). The interaction of Sex and Emotion was marginally significant, F (5, 230) = 3.24, p = .02. Post hoc tests indicated that there were no differences in how accurately each emotion was recognized by male versus female participants; rather, the pattern of responses to the six emotions showed slight differences in accuracy when male and female participants were inspected.

#### (ii) Confidence ratings

Gating studies of auditory word recognition have analyzed confidence ratings as a secondary measure of whether listeners recognized the target meaning encoded at specific gate locations (where increased confidence about correct target judgements is thought to reveal actual recognition of the underlying meaning [55], [56]. To examine these measures in the context of vocal *emotion* recognition, confidence ratings corresponding to all correct target responses were averaged at each gate interval; these data are provided in the bottom panel of Table 2, by emotion and gate. The mean confidence ratings were entered into a 6×7 ANOVA with repeated factors of Emotion (anger, disgust, fear, sadness, happiness, neutral) and Gate duration (Gate 1–7). This analysis excluded one male participant who failed to correctly recognize any of the disgust items (producing an empty cell for this analysis).

The effect of Emotion, *F* (5, 235) = 27.42, *p*<.001, *ŋ^2^* = 0.37, Gate duration, *F* (6, 282) = 136.98, *p*<.001, *ŋ^2^* = 0.74, and the interaction of Emotion × Gate duration, *F* (30, 1410) = 7.78, *p*<.001, *ŋ^2^* = 0.14, were all highly significant. Post hoc elaboration of the interaction indicated that generally, confidence ratings of each emotion tended to increase as a function of increased gate duration. At Gate 1, participants were most confident in their recognition of sadness, which significantly exceeded their confidence ratings of anger, fear, and disgust. In turn, confidence ratings of these four emotions were significantly greater than for happiness and neutral (which did not differ). At Gate 2, participants were significantly more confident when they identified sadness and anger when compared to fear, which in turn was significantly greater than for disgust, happiness, and neutral (which did not differ). In the middle portion of the utterance (Gates 3 to 5), there was a consistent dichotomy: listeners were significantly more confident when they recognized sadness, anger, and fear when compared to disgust, happiness, and neutral. At the end of the utterance (Gates 6 & 7), there were no significant differences in how confident listeners were when they recognized discrete emotions in the voice, with the exception of disgust which was always associated with lower ratings/less confidence when compared to all other emotions.

### Emotion identification points

Our first set of analyses established that the recognition of discrete emotions evolves in qualitatively distinct ways when accuracy measures/confidence ratings are examined over the course of an utterance. As the next step, we devised measures to estimate which gate interval is associated with the *isolation* of discrete emotional meanings—i.e., the probable “identification point” of each emotion within an utterance—to arrive at a description of the temporal and acoustic features which correspond to vocal emotion recognition.

Following previous methods [49], we examined the emotional responses assigned by a given participant to the seven gated versions of the same pseudo-utterance, from shortest to longest gate duration. We then pinpointed the exact gate interval at which the intended target meaning was correctly identified by the participant, and did not change at later gate durations for that stimulus. This new dependent measure, referred to here as the “emotion identification point” of the stimulus (specified as gate 1 to 7), was determined separately for each utterance when judged by each of the 48 participants (6 emotions×24 items = 144 identication points/participant×48 participants = 6912 total identification points, or 1152 data points per emotion). Following Salasoo and Pisoni [51], our scoring system allowed instances when there was only one incorrect response following at least two consecutive correct target responses in the gated series (for example, the identification point of an anger stimulus with successive responses of “neutral, anger, anger, anger, disgust, anger, anger” was scored as gate 2). As expected in tasks involving emotion judgements, there were many cases which did not lead to stable identification of the intended emotional target, and these were scored as errors.

#### (i) Frequency distribution

The distribution of (correct) identification points for each emotion at each of the seven gate intervals, as well as the frequency of errors per emotion (i.e., cases which did not lead to stable identification of the target emotion by Gate 7), are furnished in Table 4. The location of emotion identification points within an utterance varied notably by emotion type, although the most frequent location generally occurred after listening to only Gate 1 (sadness, neutral) or Gate 2 (anger, fear, happiness). Interestingly, this means that for sad and neutral utterances, a substantial portion of the stimuli were correctly differentiated from the other emotional meanings after hearing only the first, unstressed syllable of the utterance (emotion identification points occurring at Gate 1: sadness = 50% and neutral = 43% of all correctly identified exemplars). Emotion identification points for anger and fear occurred predominantly in the first three syllables of the utterance (Gates 1 to 3); when combined, the first three gate intervals accounted for 70% of correct anger identifications and 66% of correct fear identifications. In contrast, happiness and disgust were rarely identified after Gate 1 and showed a more even distribution of identification points throughout the utterance. Disgust tended to be identified much later in the utterance than the other emotions (most frequently at Gate 7).

**Table 4:** Frequency of emotion identification points observed at each gate duration interval of the utterance and the frequency of errors observed for each emotion (n = 1152 total observations/emotion).

	Gate Duration (# syllables)								
Emotion	G1	G2	G3	G4	G5	G6	G7	Total correct	Total errors
Anger	262(28%)	270(28%)	132(14%)	111(12%)	51(5%)	57(6%)	62(7%)	945	207
Disgust	28(3%)	91(11%)	105(12%)	119 (14%)	120(14%)	153 (18%)	232(28%)	848	304
Fear	185(18%)	285 (27%)	221 (21%)	129 (12%)	90 (8%)	71 (7%)	74 (7%)	1055	97
Sadness	530(50%)	205 (19%)	116 (11%)	76 (7%)	42 (4%)	44 (4%)	48 (5%)	1061	91
Happiness	39(4%)	195 (19%)	156 (15%)	189 (18%)	190 (18%)	145 (14%)	126 (12%)	1040	112
Neutral	453(42%)	221 (21%)	145 (14%)	87 (8%)	58 (6%)	53 (5%)	45 (4%)	1062	90

Emotion identification points refer to the gate at which the correct target response was first recognized and did not change at longer gate intervals.

The frequency of *errors* (i.e., instances when the target emotion could not be correctly identified by the final gate of the utterance) also varied by emotion type, with the greatest number affecting disgust (26% errors) and anger (18% errors). Nonetheless, stable emotion identification points could be calculated for the vast majority of responses: 82% of all responses for anger (945/1152 observations), 74% for disgust (848/1152), 92% for fear (1055/1152), 92% for sadness (1061/1152), 90% for happiness (1040/1152), and 92% for neutral (1062/1152). Thus, analyses which characterize the temporal and acoustic features associated with emotion identification points represent an average of between 848 and 1062 individual stimulus values depending on the emotion type inspected.

#### (ii) Temporal characteristics

As noted earlier, global differences in utterance duration/speech rate play an important role in how speakers express emotion, and accordingly, the mean duration of linguistically identical gates presented in the experiment varied naturally by emotion type (review Table 1). These time differences are not captured when describing emotion recognition as a strict function of the number of syllables presented to listeners. To relate emotion identification points to the actual *time* needed to recognize discrete emotions, the gate value representing each emotion identification point in our data was individually replaced with the actual duration of the corresponding stimulus gate, in milliseconds. The new, duration-corrected values provide an exact sense of how much time listeners were allowed to process vocal attributes of an utterance when this promoted accurate recognition of the emotional target (without subsequent changes at longer gate intervals), and whether this varied by emotion type.

**Figure 2:**
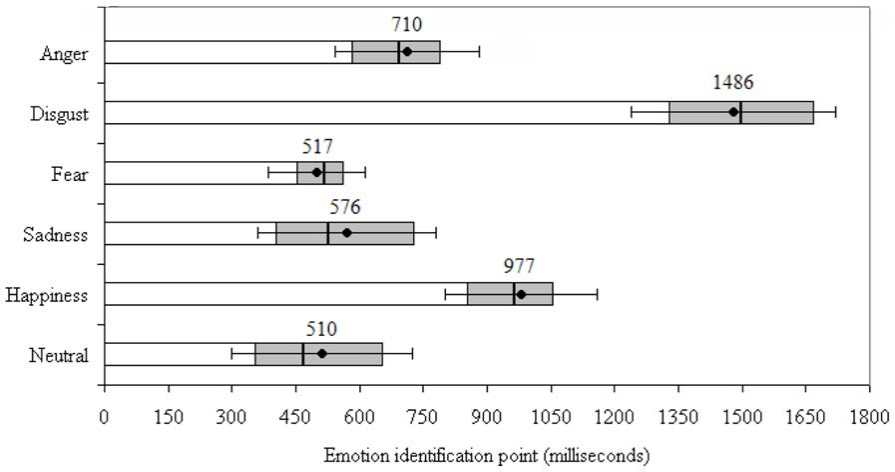
Mean recognition point for utterances conveying each emotion. Error bars refer to the standard deviation around the mean, and shaded bars refer to the 25^th^ and 75^th^ percentile within the distribution of identification points for each emotion (minimum of 848 and maximum of 1062 data points calculated per emotion category).

A one-way ANOVA with repeated measures on Emotion (anger, disgust, fear, sadness, happiness, neutral) was run on the emotion identication point measures expressed in milliseconds (ms). As this analysis included items which yielded a correct target response, the male participant who responded incorrectly to all disgust items was again omitted. The Emotion effect was highly significant, *F* (5, 230) = 194.19, *p*<.0001, *ŋ^2^* = 0.81. Post hoc Tukey's tests performed on the cell means revealed that emotion identification points for neutral (*M* = 510 ms, *SD* = 206), fear (*M* = 517 ms, *SD* = 120), and sadness (*M* = 576 ms, *SD* = 205 ms) occurred significantly earlier following speech onset, than the identification points for anger, happiness, and disgust. Furthermore, anger (*M* = 710 ms, *SD* = 174) could be identified from significantly less speech information than happiness (*M* = 977 ms, *SD* = 187). Emotion identification points for disgust (*M* = 1486 ms, *SD* = 258) occurred significantly later after speech onset than for all other emotions. These relationships are displayed in Figure 2 which displays the approximate time window for recognizing discrete emotions from vocal cues, when identical pseudo-utterances conveying six emotion types are gated by syllable duration.

#### (iii) Other acoustic characteristics

In a final set of analyses, we sought to specify other major acoustic parameters associated with vocal expressions of each emotion type at their established identification points. For each stimulus, we again replaced the gate value representing the emotion identification point for that item with corresponding acoustic measures, independently for each of the 48 listeners. The acoustic parameters of interest were: mean fundamental frequency (f0Mean, in Hertz), measured from sentence onset to the emotion identification point; fundamental frequency variation (f0Range, in Hz), calculated as the maximum – minimum f0 measured from sentence onset to the emotion identification point; and speech rate (SpRate), calculated as the number of syllables per second at the emotion identification point. These acoustic parameters are considered central features which differentiate emotions expressed through prosody [29]. While speech rate could be meaningfully compared across items and speakers without further normalization, f0 measures were normalized prior to statistical analysis to mitigate individual speaker characteristics unrelated to emotion (e.g., male/female voices). Following [16], raw f0 measures were standardized separately for each speaker using the average minimum f0 of all neutral utterances produced by that speaker as a single anchor point. This approach allows emotional utterances to be characterized across speakers and recording sessions in reference to a stable neutral baseline, where a normalized value of “1” always reflects a doubling of the speaker's characteristic resting frequency in a particular emotional condition, when compared to the neutral condition for that speaker (see [16] for further details).

**Figure 3:**
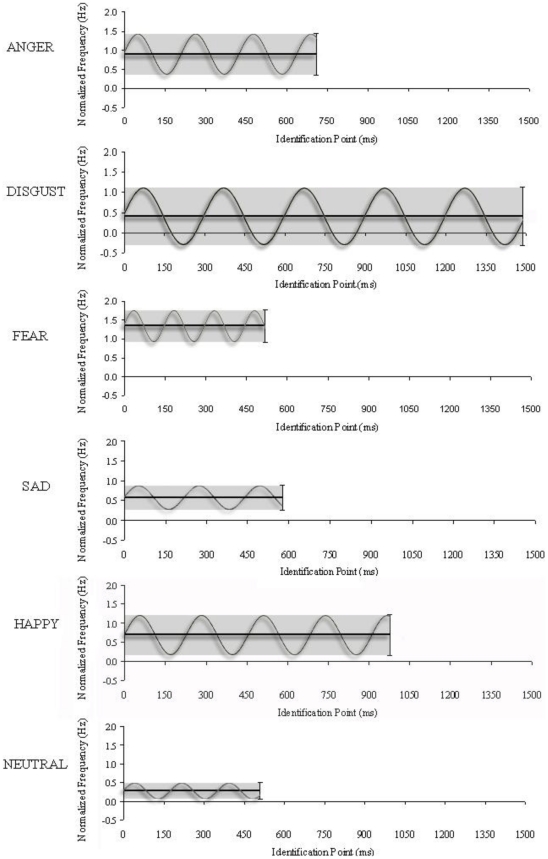
Illustration of “prototypical” acoustic features associated with utterances measured at their emotion identification point. Each period of the sine wave represents the average duration of a syllable when produced to express each emotion, which are shown up to the average emotion identification point for the corresponding emotion. The horizontal line refers to the (normalized) f0Mean of utterances, and the waveform amplitude (shaded region) refers to the f0Range, at the corresponding emotion recognition point.

Three separate one-way ANOVAs examined how each normalized acoustic measure differed as a function of the six emotion types at the exact time when each emotion was recognized, at their emotion identification point. The effect of Emotion was highly significant for each acoustic parameter investigated: f0Mean, *F*(5, 230) = 3872.32, *p*<.0001, *ŋ^2^* = 0.99; f0Range, *F*(5, 230) = 188.29, *p*<.0001, *ŋ^2^* = 0.80; and SpRate, *F*(5, 230) = 441.16, *p*<.0001, *ŋ^2^* = 0.91. Post hoc elaboration of the Emotion effect showed that global positioning of a speaker's f0Mean differed significantly for all six emotion types at the point of emotion recognition; from highest to lowest f0Mean, the pattern was: fear > anger > happiness > sadness > disgust > neutral. In the case of f0Range, post hoc tests revealed that disgust exhibited significantly greater f0 variation that anger and happiness (which did not significantly differ); moreover, expressions of disgust, anger, and happiness demonstrated significantly greater f0 variation than fear, which exhibited significantly more f0 variation than sadness and neutral expressions (which displayed the least f0 variation of all emotion types). Finally, for speech rate our data show that fear was expressed more quickly, and disgust was expressed more slowly, than all other emotions at their emotion identification point. After fear, neutral expressions were spoken significantly faster than anger, happiness, and sadness, none of which differed significantly in speech rate. Figure 3 provides a schematic illustrating the time course for vocal emotion recognition, along with prototypical acoustic properties associated with this ability, for the six emotion types at their identification point.

## Discussion

### Recognition of vocal emotion expressions over time

Our initial goal was to document how the recognition of discrete vocal emotions evolves over the course of an utterance, and to compare these patterns across emotions. Several conclusions can be drawn from the accuracy data and corresponding confidence ratings of our listeners. Most generally, the findings confirm that recognition of vocal emotion attributes in speech builds incrementally over the course of an utterance, for all six emotion types; this led to increased target recognition accuracy, and higher confidence ratings, when emotional judgements were made at longer gate intervals. This broad pattern implies that listening to longer portions of an utterance tends to facilitate processes of explicit recognition and the ability to categorize the meaning of emotional prosody [54], [55], [58], at least when emotional meanings are unambiguous and do not shift over the course of the utterance.

Of greater importance, there were marked, emotion-specific patterns in recognition accuracy directly from the beginning of the utterance (Gate 1), highlighting differences in the rate at which recognition of each emotion improved from one gate to the next (and sometimes the gate interval where recognition first began to improve). Specifically, when accuracy measures were corrected for individual response bias, our data reveal that *anger*, *sadness*, *fear*, and *neutral* expressions were recognized at comparable, increasing accuracy levels at all gates throughout the utterance, immediately from sentence onset (Gate 1). The sole exception to this clustering pattern pertained to *fear*; after Gate 4, *fear* was always recognized significantly better than all other emotion expressions. In most instances, recognition of anger, sadness, fear, and neutral expressions increased significantly as each syllable of the utterance was added between Gate 1 and Gate 4 (and often longer).

The temporal unfolding of *happiness* and *disgust* on recognition performance was entirely distinct. In general, both of these emotions were recognized more poorly from the voice at most time intervals, approximating chance accuracy levels when listeners heard only one or two syllables of an utterance (Gates 1–2). However, when utterances are gated, our data qualify that happiness recognition increases significantly over the entire course of an utterance (between all seven gate intervals); in fact, by Gate 6, there were no statistical differences in the accuracy or confidence ratings of listeners when judging *happiness*, *anger*, *sadness*, and *neutral* expressions (although *fear* was always more accurate at long gate intervals). Thus, while our findings again show that *happiness* tends to be harder to detect from speech prosody than most basic emotions [13], [65], [66], [67], they also demonstrate that recognition of this emotion improves steadily as more of the utterance is encountered, and is ultimately comparable to other emotions when vocal expressions are relatively long (6–7 syllables).

This pattern contrasts with *disgust*, which was recognized with the least accuracy and confidence from vocal cues at *all* speech duration intervals (uniquely so after Gate 2). This result, which is well documented in the literature [26], [45], [65], [68], seems to reflect not only inferior but a *slower* ability to recognize vocal attributes of disgust during speech processing. Here, we noted significant improvements in disgust recognition only between Gates 4–7, whereas recognition of all other emotion types improved significantly from the very beginning of the utterance, between Gates 1–2. This suggests a unique time course for disgust recognition in speech, which requires extended analysis of vocal cues before it can be accurately detected. Quite likely, naturally occurring expressions of disgust are more typical in the form of affective bursts (e.g., “yuck” [69], [70]), and/or may be better conveyed through visual rather than vocal cues [26]. If true, this could partly explain why listeners find it difficult to recognize disgust when emotionally-inflected pseudo-utterances are presented, as shown here and in previous reports [26], [45]. Nonetheless, our data emphasize that if listeners are given enough time, they are ultimately capable of recognizing disgust at high accuracy levels based only on prosodic cues of an utterance, since our participants achieved a mean accuracy score of almost 70% in our G7 condition (where chance performance was approximately 17%).

Although previous studies do not allow detailed comparisons about how emotion recognition unfolds over the course of an utterance, they have invariably reported emotion-specific differences in recognition accuracy when full utterances are presented to listeners, which corresponds to Gate 7 in our experimental design. Here, our data corroborate that certain emotions can be recognized significantly better than others from the voice when evaluated in forced-choice experiments [12], [14], [16], [43], [45]. Our findings may be considered robust as they reflect the *unbiased* accuracy of our 48 listeners, who judged a larger number of items representing each emotion than most previous behavioural studies, using stimuli which are perceptually validated in the literature [16]. Since our items were selected based on their perceptual properties when full utterances were presented in a similar, forced-choice validation study, the emotion-specific differences we observed at Gate 7 are partly predicted by our methods for stimulus selection (e.g., the disgust expressions showed less consensus in our validation study that other emotions, which was replicated in the gating experiment). However, this factor cannot account for all patterns observed at Gate 7, such as why *fear* was recognized most accurately from the voice, nor does it inform patterns which reflect the evolution of discrete emotion recognition as a function of gate duration. The broader significance of emotion-specific patterns in the accuracy data is elaborated further in the General Discussion.

Finally, examination of both raw (biased) and unbiased accuracy measures highlights listener biases which affect vocal emotion processing, especially at early gate intervals. When listeners were exposed to very short speech intervals (Gates 1–3), they very frequently assign an emotional value of *sadness* or *neutral* to these stimuli (review Table 2). However, correcting for individual preferences in response category usage eliminates the apparent advantage for detecting *sadness* and *neutral* as spoken language first begins to unfold; rather, this appears to reflect a systematic response bias dictated by the lack of acoustic variation which is naturally observed in the first 2–3 gate intervals for all vocal expressions. As shown in Table 1, critical acoustic parameters for understanding emotion, especially pitch variation (f0Range), require time to emerge but change rapidly in the intervals between Gates 2–4. In the absence of emotionally distinctive, long-term changes in these parameters when speech intervals are too short, it seems that listeners identify the speaker as sounding sad or neutral because these emotions are actually defined by a lack of acoustic variation along several dimensions, especially for pitch/f0 [26], [29]. These data serve to elaborate the claims of Cornew et al. [55] who only studied neutral (and not sad) stimuli in their gating study, suggesting that responses at early gate intervals do not reflect a simple bias for recognizing neutral prosody. In the case of *neutral* expressions, listeners may also be adopting a default, guessing strategy when overt cues to emotion cannot be recognized. We found that listeners were significantly less confident when they correctly recognized *neutral* expressions, but not *sad* expressions, at the early gate intervals; this implies that while listeners could not detect explicit emotional qualities from speech segments which were very short, they were simultaneously unsure as to whether the speaker *intended* to speak in a neutral tone of voice. These patterns stress that, despite controlling for individual response bias in our analyses, accuracy measures index a variety of strategies commonly used by listeners when *categorizing* stimuli in the forced-choice response paradigm.

### Emotion Identification Points

The second major question posed in this study was: where is the approximate identification point for each emotion as listeners process an utterance, and does the time course for recognition differ by emotion type? And what major acoustic differences characterize utterances at the very point where discrete emotions are reliably differentiated? Answering these questions will serve as a foundation for new studies which investigate the timing of emotion recognition during speech processing, and which establish what acoustic parameters guide recognition processes.

Our results provide strong indications that the time needed for listeners to recognize discrete emotions from prosody varies significantly by emotion type. When all emotion identification points (totaling nearly 7 000) were expressed as the time that listeners were actually exposed to speech, there were marked differences in how quickly emotions were recognized from prosody in otherwise identical utterances. Fear, sadness, and neutral expressions were recognized in the shortest time interval, with accurate recognition of these emotions emerging, on average, in the period of 500–600 milliseconds following speech onset. In broad terms, our observations extend data suggesting that discrete emotional meanings conveyed by prosody are implicitly registered in memory in the 300–600 ms time window [39], [40], [42], specifying that this knowledge is available for conscious processing somewhat later in this time period (beginning around 500–600 ms after speech onset). Also, our data for neutral stimuli correspond well with those reported by Cornew et al. [55], who reported a mean emotion identification point of 444 ms when sentences were gated in 250 ms intervals (versus a mean of 510 ms here when utterances were gated by syllable). Interestingly, explicit recognition of emotional faces also appears to begin in the 500–600 time window according to recent data [71], [72].

However, similar to what has been reported for faces, not all vocally-expressed emotions were recognized in this early time window; anger took approximately 700 ms to recognize, happiness took 1000 ms, and disgust took almost 1500 ms on average. Given patterns in our accuracy results, it is not surprising that happiness and especially disgust required significantly more exposure to speech than the other emotion types for accurate recognition, although this difference in timing remains highly marked (e.g., identification points for disgust were nearly three times longer than for fear). To some extent, our conclusions about the time needed to recognize emotions from the voice are dictated by how we constructed our gates, which were defined by syllable duration rather than precise time increments. As the mean syllable duration varied systematically by emotion type, gating utterances by syllable duration may have somewhat inflated the time needed to recognize certain emotional expressions (particularly those with long syllable durations, such as sadness and disgust). For example, if the precise emotion identification point fell shortly after the boundary of two syllables, our procedures would have nonetheless added 200–400 ms to the estimated identification point for that item (depending on the emotion), when the precise identification time actually fell in the early portion of the gate at which the emotion identification point was defined. These factors could have exaggerated our timing measures to some degree.

However, it is unlikely that this factor contributed in a major way to our findings; we observed that fear and sadness both required the *least* amount of acoustic information to recognize, despite the fact that syllable durations for fear tended to be shortest, and sadness tended to be one of the longest, on average. It should be noted that Cornew et al. [55] also reported that anger and happiness take relatively long to isolate and categorize in speech (*M* = 723 ms and 802 ms, respectively), in agreement with our findings for these two emotions. Still, the absolute timing measures we report for each emotion should be viewed as estimates at this stage of analysis until further studies can elaborate on these findings. In contrast, the *relative* differences we observed in how quickly discrete emotions are explicitly recognized were large and robust for these data; this provides the most compelling evidence to date that discrete emotions in the voice unravel to listeners at different rates, and are associated with a distinct time course during speech processing.

Our report represents a comprehensive example of how identification points can be calculated in the study of emotional prosody recognition. In so doing, our data provided a unique opportunity to relate the recognition of each stimulus to the precise acoustic features available to listeners at their point of recognition. Previous investigations which have compared emotion recognition with underlying acoustic features of speech (e.g., [16], [26]) have concentrated on whole utterance measures, which do not directly correspond to the *time point* where recognition was established which we report here. We observed marked differences in f0 parameters of speech at the emotion recognition point: fear exhibited a very high f0Mean and moderate f0 variation, whereas disgust displayed a very low f0Mean and high f0Range. Sadness and neutral both exhibited a moderate to low f0 mean as well as low f0 range (sadness was significantly higher than neutral on both measures). Anger and happiness both exhibited moderate settings of f0Mean and f0Range (with anger showing a significantly higher f0Mean than happiness). Differences in emotion recognition were further informed by speech rate: fear was produced very quickly at its emotion identification point, whereas disgust was produced with a slower speech rate than the other emotions. Neutral expression were produced significantly faster than all emotions except fear, whereas anger, happiness, and sadness were all produced at a similar, moderate speaking rate at their recognition point.

The acoustic data we report, which correspond closely to the estimated point of emotion recognition, fit acoustic descriptions of the six emotion expression types when whole utterances are measured (see [29] for an overview). Given this resemblance, it is possible that our timing measures reflect the point where acoustic patterns first begin to display prototypical or ‘modal’ properties referring to each emotion, allowing accurate recognition of their meaning (and once manifest, these patterns remain largely unchanged throughout the duration of the utterance, as shown by data in Table 1). Certainly, our measures underscore that *multiple* acoustic parameters contribute simultaneously to how listeners “isolate” discrete emotions in speech, as each of the acoustic parameters of interest differentiated at least four of the emotion types, but in different ways for each parameter. Uniquely, all six emotion types could be differentiated at their recognition point based on the speaker's adopted f0Mean, indicating that perceived voice pitch over time acts as a particularly critical indicator of a speaker's emotion state to listeners [26], [30], [63]. As more research of this nature is undertaken, future studies will more precisely show how emotion recognition is influenced by combined changes in these and other, unexplored acoustic parameters.

Finally, as identification points are rarely calculated in gating studies of vocal emotion processing, our data have methodological implications for future work. While allowing estimates of timing, defining emotional prosody recognition as the frequency of emotion identication points occuring at each gate leads to a characterization of how recognition accuracy unfolds that is distinct from both the raw and unbiased hit rates (although the proportion of correct emotion identification points computed for each emotion closely mirrors the raw accuracy scores of the 48 participants at Gate 7). However, like the raw accuracy data, computing the location of emotion identification points in an utterance as we did here would be influenced to some extent by participant response biases; this is why, for example, emotion identification points for sadness and neutral occurred most frequently at Gate 1; as argued above, these effects are partly explained by response biases to choose “neutral” or “sad” when listeners are presented very short auditory stimuli which contain little acoustic variation in the shortest gate intervals.

### General Discussion

Intuitively, few have questioned the notion that vocal emotion expressions, which encode meaning via patterned cue sequences in speech, require a certain amount of time for listeners to accurately recognize. It is therefore curious that few concerted attempts have been made to empirically validate this assumption, by precisely demonstrating *how much* vocal information listeners need to consciously recognize discrete emotions as spoken language unfolds. One of the unique insights uncovered by our data is that there is a distinct time course associated with the recognition of basic emotions expressed in the vocal channel of speech. Using a gating paradigm, our data show that basic emotions encoded in the voice unfold in qualitatively distinct ways and at different rates, yielding marked emotion-specific patterns in recognition accuracy as accumulating acoustic evidence of the utterance is revealed.

Until recently, much of the literature which informs the nature of emotion recognition from the voice, and also the face, has used behavioural methodologies and the forced-choice response format (e.g., [45], [73]). Forced-choice tasks, by their nature, characterize ‘recognition’ broadly; here, our task would have tapped early, automatic procedures for analyzing the acoustic input, and for activating initial representations of emotion based on the acoustic evidence [17]. These operations, which may be *uniquely* registered by many on-line tasks of implicit processing of vocal emotion cues (e.g., [7], [39]), are believed to preferentially engage mid- and posterior portions of the superior temporal gyrus/sulcus, respectively, at the neural level of analysis [17], [74]. In addition, our gating measures would index operations which promote explicit cognitive evaluation of vocal emotion cues in relation to the contents of emotional memory, and strategic mapping of this information onto verbal labels that refer to emotion categories. These latter procedures, which are necessary to execute explicit emotion judgements in a goal-directed manner, seem to recruit inferior frontal regions of the brain to arrive at a more complex, cognitively-elaborated sense of the meaning of vocal emotion expressions [75], [76]. These latter operations are most susceptible to methodological factors, such as the number or type of emotional response alternatives in the experiment, social attributes of the participants, and other task-related demands [77], [78].

The specific emotions examined in this study—anger, disgust, fear, sadness, and happiness—are all believed to have evolved unique signal functions in human communication, which govern how they are encoded and decoded [11], [62]. Until now, evidence that vocal expressions of basic emotion are discrete in their temporal processing characteristics as speech is consciously analyzed, affecting the time course of processes which lead to the recognition of specific emotions, has not been conclusively demonstrated. Our data establish that many emotions can be recognized accurately from the voice after hearing only one or two syllables of an utterance, although the actual amount of time needed is highly variable by emotion type. When defined by emotion identification points, recognition began to emerge, on average, in the time window of 500–600 ms (for fear, sadness, and neutral stimuli), but extended to 1000 ms and well above (for happiness and especially disgust).

Since our timing measures partly reflect how well listeners strategically evaluate the emotional significance of a temporally-unfolding acoustic representation, they serve to elaborate, but do not contradict, what we already know about the timing of early, *automatic* processes which act on an emotion stimulus. According to appraisal theories, incoming events are rapidly appraised to code the stimulus for its valence, urgency, significance to the organism, and other affective dimensions (see [79] for a recent summary). The time course of early evaluative processes can be indexed by sensitive, on-line measures with fine temporal resolution, such as ERPs. In general, it seems that a preliminary analysis of the perceptual/structural features of emotional expressions, which allow their emotional *salience* to be detected (i.e., as emotional or non-emotional), occurs within 200 milliseconds following stimulus onset (yielding modulation of the P200 component for vocal emotions [38] and faces [80], [81], [82]). Further perceptual *and* early semantic analysis of the meaning of emotional expressions appears to occur in the 220–300 ms time window, where early negativities begin to show modulation linked to discrete emotional expressions when compared to neutral expressions [40], [82]. Evidence that the discrete emotional value of the expression is implicitly detected occurs approximately 400 ms following stimulus onset, based on evidence of N400 modulations to emotional mismatches involving speech stimuli [38], [39] and facial expressions [82], [83]. If proven correct, this timeline underscores that high-order use of emotional information, such as the ability to consciously evaluate the contents of emotional representations held in memory for recognition and naming, should be problematic or impossible prior to 400 ms of information processing [17]. As well, one might expect implicit priming of an emotional target stimulus by congruent vocal cues to be absent or unstable if vocal primes endure less than 400 ms, as has been reported recently [39], [42], [84].

This description is in alignment with our observation that certain emotions are explicitly recognized and “isolated”, on average, in the 500–600 ms time window (possibly sooner, given that our gating technique led only to approximate time measures). Interestingly, response times for recognizing facial expressions of emotion in the forced-choice task seem to fall in a similar time window, ranging from 544 ms (happiness) to 669 ms (contempt) following face onset [72]. Since emotion-specific knowledge about vocal expressions is presumably activated “on-line” around 300–400 milliseconds following speech onset, our data show that conscious appraisal of these cues for naming can be accomplished reliably with very little additional acoustic information for certain emotions (fear, sadness, and possibly anger). On the other hand, some emotions require protracted exposure and analysis before they can be properly identified in speech (happiness, disgust).

From a biological and evolutionary perspective, our data supply further indications that negative emotions, which signal threat, aggression, and loss, are given precedence by the neurocognitive system, allowing individuals to quickly respond in an appropriate manner to an undesirable (vocal) stimulus [85], [86]. The observation that *fear* was recognized faster, and ultimately better, than other vocal expressions of basic emotion is noteworthy [65], [66]. There are now well-defined neural systems, action tendencies, and cognitive responses associated with aversive or threatening stimuli, such as facial and vocal expressions of fear and anger [87], [88], [89]. The urgency to respond to fear-inducing stimuli, and the fact that vocal signals of fear can be highly salient in the absence of joint visual attention, may explain why these expressions are detected very rapidly in the vocal channel, even when conscious evaluation is required.

Since fearful voices are also highly distinctive in their acoustic-perceptual form—exhibiting a higher mean pitch and faster speech rate than other emotions [16], [26]—it is possible that these expressions are simpler to recognize at the perceptual level of analysis, which promotes faster and more accurate detection of fear in many processing environments (including the gating paradigm). The same reason could explain why sadness, which exhibits a distinct *lack* of acoustic variation and relatively slow speech rate, is routinely recognized with great accuracy in speech [26], [43], [67], and as demonstrated in this study, based on minimal acoustic evidence. The idea that low-level physical characteristics of fearful and sad expressions are more salient in the voice, allowing their meanings to be detected relatively quickly, will require further study; this hypothesis resembles similar explanations for the “happiness advantage” observed in studies of emotional face recognition (see [90] for a discussion).

In contrast to fear and sadness, anger took approximately 200 ms longer to recognize in our study. Since detecting anger is also a matter of biological urgency, and these expressions are typically recognized very well from the voice [26], [43], [48], the delay in our timing measures in relation to fear/sadness may have been influenced by the form of anger encoded by our vocal stimuli. The angry utterances presented in this study conveyed “cold anger” (frustration) rather than “hot anger” (rage), and thus represented a less intense form of anger with somewhat different vocal attributes (see [91] for comparative data). Since differences in arousal play a role in how well anger is recognized [26], [68], this factor could explain why extended cognitive analysis of angry expressions in this study was necessary for our listeners, leading to later recognition points for this emotion. This claim can be tested by manipulating the intensity of emotional expressions in future gating studies.

Considerably more time was needed for listeners to recognize vocal expressions of happiness (∼1000 ms) and disgust (∼1500 ms), which could relate to several factors. For happiness, despite this being the only positively-valenced emotion in our study, the lack of any advantage to recognize happy expressions accurately is predicted by the literature [12], [13], [65], [66], although our data show that these difficulties extend to slower *speed* of recognition [55]. Nonetheless, the emotional meanings of happy expressions seem to be registered in memory enough to produce category-specific priming effects, and modulation of the N400 component, after listening to prime stimuli lasting only 400 ms [39] or 600 ms [42], preceding a congruent or incongruent facial expression. Put together, this implies that happiness is recognized implicitly like other emotions, but that conscious evaluation of happy cues in speech requires prolonged exposure and greater analysis when compared to most basic emotions. It should be explored whether vocal cues signifying happiness require greater cognitive analysis because there are actually different “kinds” of happiness or positive emotions (e.g., contentment, amusement, achievement, etc.) which can be discretely recognized at the stage of conscious processing, each with a distinct acoustic signature [62], [92]. Also, based on evidence of how nonverbal emotional vocalizations are categorized, it is possible that negative emotions in the voice are recognized pan-culturally, whereas positive emotions are communicated with culture-specific signals [92]. If these principles govern how emotions are conveyed in the context of speech, our data could exemplify that speakers provide acoustic cues to the listener in a more localized manner, perhaps at the end of an utterance [27], to mark their positive disposition and/or affiliative intentions to the listener.

Finally, in the case of disgust which is a negatively-valenced “defensive” emotion, our data showcase that these expressions are recognized very slowly (and with uncertainty) in speech. There is previous evidence of specific attentional biases related to disgust; for example, participants primed with disgust-related stories demonstrated slower responses in a Stroop task [93], or had difficulties disengaging from disgusting words [94]. An emotion-specific attention bias which tends to delay behavioural responses to disgusting stimuli could partly contribute to our findings. Alternatively, it is likely that there are asymmetries in how well disgust (and other emotions) are conveyed in specific communication channels; signals of disgust seem to be more salient in the face [45] or when communicated by nonverbal vocalizations, or vocal emblems (such as “eackk” or “eew”, see [69], [70]. The fact that disgust was recognized slowly in our study may thus partly reflect the atypicality of encountering disgust through isolated vocal cues in speech, leading to difficulties for many listeners.

Overall, our new timing measures expand a growing database which argues that vocal expressions of basic emotion possess discrete acoustic-perceptual properties [13], [16], activate category-specific knowledge in emotional memory [7], [34], [39], [95], [96], [97], and are processed by partially distinct neurocognitive mechanisms [36], [98], [99]; [ cf 74]. Our investigation newly establishes that processes leading to the explicit recognition of anger, disgust, fear, sadness, and happiness are also associated with a unique time course. This report serves as a foundation for future studies which clarify how vocal emotion expressions evolve over time, and why the recognition of basic emotions unfolds in a temporally distinct manner in speech. At a methodological level, our study reinforces the utility of auditory gating as an approach for studying emotions in speech and for inferring when vocal emotion recognition occurs (in reference to their “emotion identification point”). As different investigative approaches are combined to pinpoint when basic emotions are recognized with even greater precision, the role of socio-cultural factors in vocal emotion processing, and their influence on timing, will also need to be addressed [34], [100], [101].
